# Dried Brown Seaweed’s Phytoremediation Potential for Methylene Blue Dye Removal from Aquatic Environments

**DOI:** 10.3390/polym14071375

**Published:** 2022-03-28

**Authors:** Abdallah Tageldein Mansour, Ahmed E. Alprol, Khamael M. Abualnaja, Hossam S. El-Beltagi, Khaled M. A. Ramadan, Mohamed Ashour

**Affiliations:** 1 Animal and Fish Production Department, College of Agricultural and Food Sciences, King Faisal University, P.O. Box 420, Al-Ahsa 31982, Saudi Arabia; 2 Fish and Animal Production Department, Faculty of Agriculture (Saba Basha), Alexandria University, Alexandria 21531, Egypt; 3 National Institute of Oceanography and Fisheries (NIOF), Cairo 11516, Egypt; ah831992@gmail.com; 4 Department of Chemistry, College of Science, Taif University, P.O. Box 11099, Taif 21944, Saudi Arabia; k.ala@tu.edu.sa; 5 Agricultural Biotechnology Department, College of Agriculture and Food Sciences, King Faisal University, P.O. Box 420, Al-Ahsa 31982, Saudi Arabia; helbeltagi@kfu.edu.sa; 6 Biochemistry Department, Faculty of Agriculture, Cairo University, Giza 12613, Egypt; 7 Central Laboratories, Department of Chemistry, King Faisal University, P.O. Box 420, Al-Ahsa 31982, Saudi Arabia; kramadan@kfu.edu.sa; 8 Department of Biochemistry, Faculty of Agriculture, Ain Shams University, Cairo 11566, Egypt

**Keywords:** *Sargassum latifolium*, methylene blue dye, adsorption, water pollution, equilibrium isotherm

## Abstract

The dried form of the brown seaweed *Sargassum latifolium* was tested for its ability to remove toxic Methylene Blue Dye (MBD) ions from aqueous synthetic solutions and industrial wastewater effluents. In a batch adsorption experiment, different initial concentrations of MBD (5, 10, 20, 30, and 40 mg L^−1^), sorbent dosages (0.025, 0.05, 0.1, 0.2, 0.3, 0.4, and 0.5 g L^−1^), contact time (5, 10, 15, 30, 60, 120 min), pH (3, 5, 8, 10, and 12), and temperature (30, 40, 50, 60 °C) were observed. Dried powder of *S. latifolium* was characterized before and after adsorption of MBD using different techniques, such as FTIR, SEM, UV visible spectral examination, and BET techniques. The BET surface area suggests the formation of *S. latifolium* was 111.65 m^2^ g^−1^, and the average pore size was 2.19 nm. The obtained results showed that at an MBD concentration of 40 mg L^−1^, the adsorption was rapid in the first 5, 10, and 15 min of contact time, and an equilibrium was reached in about 60 and 120 min for the adsorption. At the optimum temperature of 30 °C and the adsorbent dose of 0.1 g L^−1^, approximately 94.88% of MBD were removed. To find the best-fit isotherm model, the error function equations are applied to the isotherm model findings. Both Tempkin and Freundlich isotherm models could appropriate the equilibrium data, as well as the pseudo 2nd order kinetics model due to high correlation coefficients (R^2^). Thermodynamic and Freundlich model parameters were assessed and showed that the mechanism of the sorption process occurs by an endothermic and physical process. According to the results of the experiments, *S. latifolium* is a promising environmentally friendly approach for eliminating MBD from the aqueous solution that is also cost-effective. This technology could be useful in addressing the rising demand for adsorbents employed in environmental protection processes.

## 1. Introduction

A pollutant is a substance that changes the environment’s nature through chemical, biological, or physical mechanisms, resulting in contamination of water, soil, and/or air [1]. Several synthetic dyes are primary sources of high effluent pollutants produced as a result of industrial pollution [2]. Textile effluents are considered one of the most environmentally harmful pollutants due to their massive discharge volume and verity in composition, as well as the high volumes of poisonous dye that are lost through the dyeing operation [3]. Several industries, including paper, dyeing, pulp, textiles, paint, and industrial effluent, have produced over 8000 dyes, both insoluble and soluble [1]. Because of their carcinogenic and mutagenic properties, these poisonous dyes pose a threat to human health [4,5,6]. Furthermore, these toxic dyes have had devastating effects on aquatic ecosystems, as well as a significant impact on the distributions and compositions of aquatic animals, zooplanktonic, and phytoplanktonic organisms [7,8,9,10,11]. The dyes alter the characteristics of water and prevent sunlight from penetrating, decreasing photosynthetic activity [2,12,13]. With the harmful effects of several toxic dyes on ecosystem components, it is necessary to find appropriate and effective substances to eliminate these toxic dyes from industrial waste. Despite there being various physical, chemical, and biological processes available for the elimination of these toxic dyes, their efficiency still needs improvement [14,15,16,17]. Globally, the annual production of a large assortment of these toxic dyes is estimated at 1.6 million tons [18].

As a result, the developed techniques that are capable of eliminating dyes that have not adhered to tissue fibers over the process procedures and are emitted along with the effluents of such industries are a constant concern [19,20,21,22]. The chemical stability of synthetic textile dyes makes existing wastewater treatment technologies incapable of the treatment of effluents that contain these contaminants [23]. Adsorption methods have high adequacy in industrial wastewater treatment, owing to their significant capacity for eliminating organic matter and low cost [24,25,26,27]. Methylene blue dye (MBD) is one of the most extensively used industrial dyes, especially in the textile industry. C_16_H_18_N_3_SCl is the formula for MBD, a heterocyclic chemical molecule [28]. MBD appears as a dark green powder at room temperature and as a blue hue in the water [29]. MBD was first produced in 1876 as an aniline-based synthetic dye to stain cotton for the textile industry [30]. The maximum absorption of MBD was at around 665 nm [18]. Flocculation [31], coagulation [32], photocatalysis chemical precipitation [33,34], chemical oxidation [35], and ion exchange [36] are some of the treatment procedures used to remove organic pollutants from wastewater effluents.

Adsorbent refers to either adsorption or absorption and is currently one of the most widely used adsorbents. The amalgamation of a material into another material of an unlike condition is known as absorption (liquids are absorbed by gases or/and a solid is absorbed via a liquid). However, the adsorption process is a physical attachment of molecules and ions to another molecule’s surface. Sorption has several advantages, including efficiency, simplicity of operation, regeneration of the adsorbents, and processing [37]. Other advantages of the adsorption process, such as the potential for cost-effective adsorbent regeneration and less sludge generation, making it one of the most important treatment methods for water-intensive sectors like textiles and paints. The efficacy of the adsorption techniques for textile and dye wastewater treatment has been demonstrated through extensive previous work [38,39,40].

Numerous microorganisms’ biomass, for example, fungi, yeast, microalgae, seaweed, and bacteria, have been used as biological, sustainable, and low-cost efficient adsorption materials for the absorption of hazardous dyes, according to the literature [41,42,43,44,45,46]. Among all microorganisms, algal cells are the most promising, sustainable, and low-cost biomaterials for adsorptions [47]. In general, aquatic organisms are the richest sources of biologically active compounds on the planet [48,49,50]. However, amongst all aquatic organisms, algal cells are the richest sources of biomolecules, making algae a vital player in a variety of bioindustries [51,52,53,54,55,56]. Depending on the algal strain, algal cells contain proteins (EAAs), lipids (PUFA, AA, EPA, and DHA), and carbohydrates (polysaccharides, etc.) [57]. However, algal cells’ adsorption ability is linked to the cell wall’s heteropolysaccharide and lipid molecules, which contain several functional groups, for example, phosphate, carboxyl, amino, carbonyl, and hydroxyl groups [46].

Sargassum species are brown macroalgae (seaweed) found in shallow marine meadows in the tropical and subtropical regions. These are a good source of vitamins, carotenoids, dietary fibers, proteins, and minerals, as well as other bioactive components. Biologically active substances such as terpenoids, flavonoids, sterols, sulfated polysaccharides, polyphenols, sargaquinoic acids, sargachromenol, and pheophytine have also been identified in various Sargassum species. [58].

This work aimed to investigate the efficacy of the brown seaweed, Sargassum latifolium, as a suggested MBD removal substance. This dye is globally utilized in textile effluents. Characterizations of materials and understanding of the mechanisms comprised in the sorption process of dyes through the identification of functional groups were determined using different characterization techniques (SEM, FTIR, BET, and UV visible). The parameters (pH, contact time, sorbent dose, temperature, and initial MBD concentration) controlling the sorption process were examined. Moreover, equilibrium and kinetics studies were conducted using adsorption experiments. The experimental data have been used to adjust the Langmuir, Freundlich, Smith Halsey, Henderson, Tempkin, and Harkins–Jura models.

## 2. Materials and Methods

### 2.1. Brown Seaweeds (Sargassum latifolium)

Brown seaweed, *S. latifolium,* samples were collected in May 2021 from the intertidal zone of Suez Bay, Red Sea, Egypt. To remove salts and particle materials adhering to the surface, samples were first washed with seawater, then transferred to the laboratory and cleaned with tap water, followed by deionized water [59]. After sample cleaning, *S. latifolium* was sun-dried, and the obtained biomass were powdered using a grinder, sieved to gain a uniform particle size, and stored in plastic bags until further use. Approximately one gram of the brown algae powder was soaked in 100 mL of deionized water and boiled at 45 °C for 1 h to obtain the extract.

### 2.2. Methylene Blue Dye (MBD)

**Figure 1:**
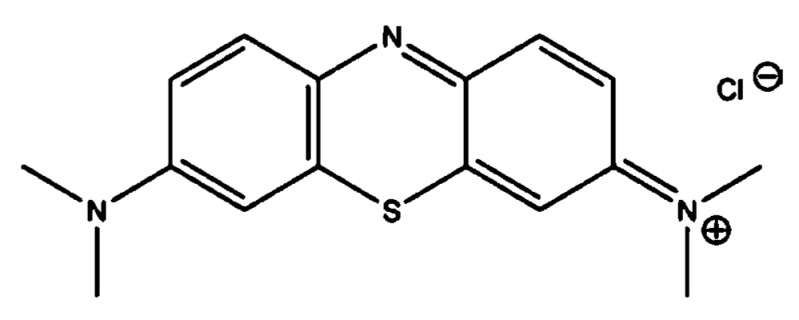
Chemical structure of the Methylene Blue Dye (MBD).

The solid form of the studied dye (MBD) was obtained from Sigma-Aldrich (Milano, Italy). MBD (Figure 1) is a cationic dye with the chemical formula C_16_H_18_N_3_SCl, molar mass of 319.85 g mol^−1^, and a maximum wavelength of 665 nm [60]. MBD has a positively charged S atom and is highly water soluble at 293 °K. The stock solutions of MBD were made via dissolving one gram of MBD in one liter of distilled water.

### 2.3. Adsorption Experimentation

The seaweed, *S. latifolium,* was utilized as a low-cost adsorbent in this investigation to remove MBD from a synthetic aqueous solution. In distilled water, dried powdered *S. latifolium* was suspended and then exposed to a vortex (Dremel, 1100-01, São Paulo, Brazil) at 10,000 rpm for 20 min [47]. Using batch ion exchange, the experiments were carried out in flasks (100 mL of distilled water, three replicates of each treatment) in a constant shaker (120 rpm) under different initial MBD concentrations (5, 10, 20, 30, and 40 mg L^−1^), adsorbent doses of *S. latifolium* (0.025, 0.05, 0.1, 0.2, 0.3, 0.4, and 0.5 g L^−1^), different contact times (5, 10, 15, 30, 60, and 120 min), temperature (30, 40, 50, and 60 °C), and pH (3, 5, 8, 10, and 12). The adsorption capacity (q_e_) and MBD elimination percentage were calculated using Equations (1) and (2), as follows [61]:

(1) $$q_{e}=\frac{\left(C_{i}-C_{f}\right)\times V}{W}$$

(2) $$\mathrm{Percentage}\;\mathrm{removal}\;\left(\%\right)=\frac{\left(C_{i}-C_{f}\right)}{C_{i}}\times 100$$

where C_i_ and C_f_ (mg L^−1^): the early concentration at the primary contact time in addition to, the final concentration of MBD at a definite time, respectively, the volume of the MBD mixture (L) is represented by V, while the weight of the dry adsorbent is represented by W (g).

### 2.4. Adsorption Isotherm Studies

The amount of MBD removed from aqueous solutions is highly dependent on the initial MBD concentrations. To measure the isotherm study, various MBD concentrations (5 to 40 mg L^−1^) were examined at constant factors of 0.01 g of *S. latifolium* adsorbents, pH 6, 30 °C, and at 150 rpm, 120 min, and mixed with 50 mL of MBD solution [62]. The data were fitted and calculated in terms of the isotherm models of Freundlich, Langmuir, Henderson, Halsey, Harkins–Jura, Smith, and Tempkin.

#### 2.4.1. The Freundlich Model

By plotting a curve of log q_e_ with relation to log C_e_, the efficiency of the Freundlich model to describe the experimental data was used to produce a slope of n and an intercept value of K_f_. By plotting the Freundlich model in logarithmic form, it is easy to linearize [63]:

log q_e_ = log K_f_ + 1/n log C_e_ (3)

Freundlich parameters K_f_ and n were calculated from the isotherm equation by Equation (3).

#### 2.4.2. The Langmuir Model

The model of Langmuir predicts uniform adsorption energies of a solute from a liquid solution onto a surface with a finite number of equal sites as monolayer adsorption with no adsorbate movement in the surface plane [64]. To estimate the highest adsorption capacity similar to complete monolayer adsorption on the sorbent surface, the Langmuir isotherm model was used. The mathematical expression represents the Langmuir model as the following equation [65]:

q_e_ = q_max_ bC_e_/(1 + bC_e_) (4)

where q_max_ (mg g^−1^) considers the highest sorption capacity in proportion to the saturation capacity, and b (L mg^−1^) shows a coefficient relating to the affinity between *S. latifolium* and dye ions. The values of q_max_ and b calculated from the intercept and slope, respectively, by plotting curve (1/q_e_) vs. (1/C_e_) yields the following linear relationship:

1/q_e_ = 1/(bq_max_ C_e_) + 1/q_max_ (5)

#### 2.4.3. The Henderson and Halsey Isotherm Models

These models are suitable for heterosporous solids and the multilayer sorption technique [66,67]. These models were calculated using the following equations:

(6) $$\mathrm{Ln}\;q_{e}=\frac{1}{n}\mathrm{Ln}\;K+\frac{1}{n}\;\mathrm{Ln}\;C_{e}$$

where: n and K are Halsey constants. Meanwhile, the Henderson model was obtained from the following equation:

(7) $$\mathrm{Ln}\left[\mathrm{nLn}\left(1{\mathrm{nC}}_{e}\right)\right]=\mathrm{LnK}+\left(\frac{1}{n}\right){\mathrm{Lnq}}_{e}$$

while: the Henderson constants are nh and Kh.

#### 2.4.4. The Harkins–Jura Model

This model explains multilayer sorption and the presence of heterogeneous pore scattering in a sorbent [68]. This model was obtained from the following equation:

(8) $$\frac{1}{q_{e}^{2}}=\left(\frac{B_{2}}{A}\right)-\left(\frac{1}{A}\right)\log C_{e}$$

where: the isotherm constants are A and B.

#### 2.4.5. The Smith Model

For heteroporous solids and multilayer adsorption, the Smith model is acceptable. This model is usually obtained from the following equation:

q_e_ = W_bS_ − W_S_ Ln (1 − C_e_) (9)

where: the Smith model parameters are W_bs_ and W_s_.

#### 2.4.6. The Tempkin Model

The Tempkin isotherm model assumes that owing to adsorbate–adsorbent interactions, a uniform distribution of binding energies up to greater binding energies characterizes the sorption process, and the heat of adsorption of each particle in the layer decreases with saturation [69,70]. The following equation was used to calculate the Tempkin model:

q_e_ = B Ln A + B Ln C_e_ (10)

where: A is the equilibrium binding constant (L g^−1^), b (J mol^−1^) is a constant that corresponds to the heat of sorption, and B = (RT/b) (J mol^−1^) is the Tempkin constant, which is connected to the heat of adsorption and the gas constant (R = 8.314 J mol^−1^ K^−1^).

### 2.5. Error Functions Tests

Various error functions were examined to find the best and most fitting model for analyzing the equilibrium data. The following models were used for the error function test [40,71].

#### 2.5.1. Fractional Error Hybrid (HYBRID)

Because it adjusts for small concentrations by balancing absolute deviation versus fractional error and is more dependable than other error functions, the hybrid fractional error function is used. The following equation represents the hybrid error (11).

(11) $$\mathrm{HYBRID}=\frac{100}{N-P}\sum {\left|\frac{q_{e,\exp}-q_{e,\mathrm{calc}}}{q_{e,\exp}}\right|}_{i}$$

where: the q_e,exp_ and q_e,calc_ denote the experimental and the calculated data (mg g^−1^), in addition to N is the number of parameters of the isotherm equation, and P the number of data points.

#### 2.5.2. Average Percentage Error (APE)

The ABE model shows a tendency or appropriateness among the predicted and experimental values of the sorption capacity used for plotting model curves (APE) and can be designed consistent with the following equation:

(12) $$\mathrm{APE}\left(\%\right)=\frac{100}{N}\overset{N}{\underset{i=1}{\sum }}{\left|\frac{q_{e,\mathrm{isotherm}}-q_{e,\mathrm{calc}}}{q_{e,\mathrm{isotherm}}}\right|}_{i}$$

The number of data points under investigation is denoted by the letter N.

#### 2.5.3. Nonlinear Chi-Square Analysis (χ^2^)

The nonlinear chi-square experiment is a statistical factor for evaluating which treatment system is best. The approach of the chi-square error, χ^2^, and model is assumed as the following equation:

(13) $$\chi^{2}=\frac{{\left(q_{e,\mathrm{isotherm}}-q_{e,\mathrm{calc}}\right)}^{2}}{q_{e,\mathrm{isotherm}}}$$

#### 2.5.4. Sum Squares of the Errors (ERRSQ)

The following Equation (14) gives the sum of the squares of the errors (ERRSQ) [72].

(14) $$\mathrm{ERRSQ}=\overset{P}{\underset{i=1}{\sum }}{\left(q_{e,\mathrm{calc}}-q_{e,\mathrm{isotherm}}\right)}_{i}^{2}$$

#### 2.5.5. Sum of Absolute Errors (EABS)

An increase in errors increases the fit, resulting in a bias toward high concentration data. EABS examinations can be evaluated [73] using the following equation:

(15) $$\mathrm{EABS}=\overset{P}{\underset{i=1}{\sum }}\left|q_{e,\mathrm{calc}}-q_{e,\mathrm{isotherm}}\right|$$

where P is the number of data points.

### 2.6. Adsorption Kinetics Studies

#### 2.6.1. Pseudo-First-Order (PFO)

The model equation of the generalized pseudo-first-order equation is given by the following equation [74].

Dq/d_t_ = K_1_ (q_e_ − q_t_) (16)

where q_e_ represents the quantity of dye adsorbed at equilibrium (mg g^−1^), q_t_ represents the number of dyes adsorbed at time t (mg g^−1^), and K_1_ represents a pseudo-first-order rate constant (min^−1^). The integrating equation was assessed [75] as follows:

Log (q_e_/q_e_ − q_t_) = k_1_t/2.303 (17)

In a linear equation, the PFO equation is given by the formula:

Log (q_e_ − q_t_) = log q_e_ − k_1_t/2.303 (18)

Log(q_e_ − q_t_) against (t) plots should show a linear relationship between k_1_ and q_e_, as measured by the slope and intercept, respectively.

#### 2.6.2. Pseudo-Second-Order (PSO)

The PSO equation is expressed as follows [76].

dq_t_/d_t_ = K_2_(q_e_ − q_t_)^2^ (19)

where: K_2_ indicates the constant of the second-order rate (g mg^−1^ min). The integrated equation is presented as follows:

1/(q_e_ − q_t_) = 1/q_e_ + K_2_ (20)

Following Ho et al. [76] and the following linear form of the pseudo-second-order equation:

t/q_t_ = 1/K_2_ q_e_^2^ + t/q_e_ (21)

Plotting (t/q_t_) versus (t) would yield a linear relationship, and the values of (t/qt) would be the values of the q_e_ from the slope and intercept can be used to calculate the K2 parameters, respectively.

#### 2.6.3. The Intraparticle Diffusion Model

The following is the intraparticle diffusion equation [14]:

q_t_ = K_dif_ t^1^^/2^ + C (22)

where C denotes the intercept, and K_dif_ (mg g^−1^ min^0.5^) is the intraparticle diffusion rate constant, which is calculated using the regression lines of the slope.

### 2.7. Characterization of Adsorbents

The morphological characterization (before and after the experiment) of the adsorbent brown seaweed, *S. latifolium,* was performed via Scanning Electron Microscope, SEM (JEOL JSM 6360, Peabody, MA, USA), followed by Fourier transform infrared (Shimadzu FTIR-8400 S, Kyoto, Japan). A UV–vis spectrophotometer (UV-2550, Shimadzu, OSLO, Kyoto, Japan) was used to evaluate the absorption spectrum of *S. latifolium* marine algae extract in the range of 200–800 nm, with deionized water as a blank.

## 3. Results and Discussion

### 3.1. Characterizations

#### 3.1.1. Functional Groups

**Figure 2:**
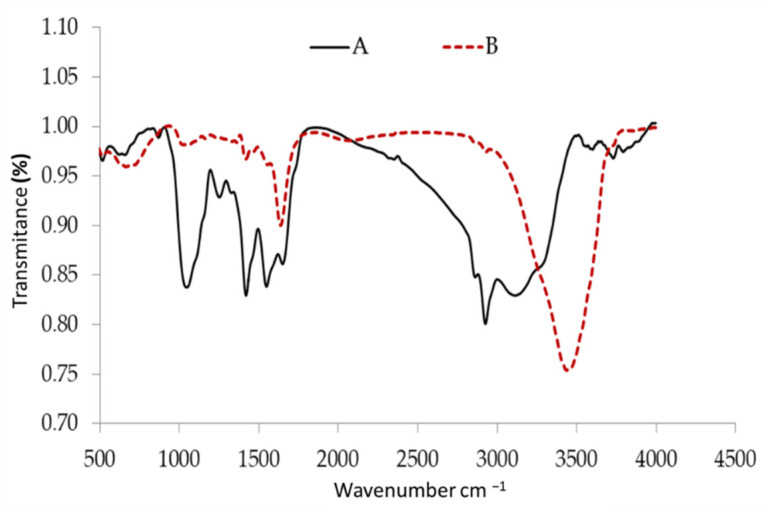
FTIR analysis of *Sargassum latifolium* before (A) and safter adsorption (B) of methylene blue dye.

The infrared spectrum of biomass in nature is shown in Figure 2. The stretching of the extension vibrations of the –NH and O–H groups was attributed to the inter- and intermolecular hydrogen bonding of polymeric compounds such as phenols or alcohols, which resulted in large and intense peaks at 3112 and 3729 cm^−1^ [77]. Similarly, the band at 2925 cm^−1^ corresponds to methylene’s symmetric and asymmetric stretch (H-C-H), whereas the peaks at 1649 cm^−1^ are C=C stretches, which could be owing to the presence of aromatic, olefinic, or N-H bending bands.

Only dye saturated biomass showed a peak, as the 1547 cm^−1^ band, indicating the presence of C=N and C=C stretch and the typical absorptions. Stretching of the sp3 C-H bond is responsible for the band at 1419 cm^−1^. The C-O stretching of acyl or phenol is related to the sharp peak at 1251 cm^−1^. The absorption band of the –OH and –NH groups at 3733 cm^−1^ changed to 3436 cm^−1^ after interaction with MBD adsorption, as shown in Figure 2. The C–H stretching vibrations of sp^3^ hybridized C in CH_3_ and CH_2_ functional groups is assigned to another peak with a shoulder at 2937 cm^−1^ and 2939 cm^−1^ [78]. CH_3_ bending and C–H stretching vibrations are responsible for the other absorption peaks, which occurred at 1419, 1467 cm^−1^, and 1361 cm^−1^, respectively. Algal cells have many functional groups on their surfaces, such as phosphate, hydroxyl, amino, and carboxylate, which are responsible for removing pollutants from various wastewaters. The accumulation of dye ions on the biopolymers of the algal surface, followed by dye migration from the aqueous phase to the biopolymer solid phase, could be the source of dye removal using algae [79].

#### 3.1.2. Surface Morphology

**Figure 3:**
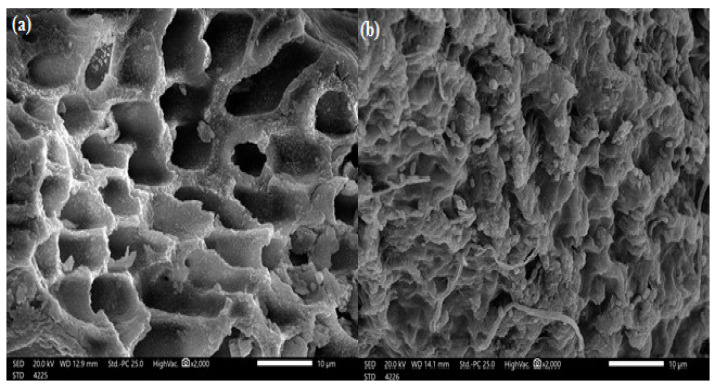
Scanning electron microscope of *Sargassum latifolium* at (**a**) before and (**b**) after adsorption of methylene blue dye.

SEM analysis of algal cells showed the surface shape of the adsorbents before and after MBD adsorption. Figure 3 presents SEM pictures of *S. latifolium* before and after dye uptake at 2000 magnifications as an example. Figure 3a shows that the adsorbent has a uniform porosity structure and that salt crystals are present on the biosorbent’s outside because of natural mineral deposition. There are also spots related to crystalline salts and the irregularity of the algal cell surface. The cross-linked micropores on the adsorbent surface may enhance the efficient uptake of the liquid electrolyte and improve the adsorbent’s ionic conductivity. In Figure 3b there are significant changes to the surface morphology of the adsorbents, as well as the formation of separate aggregates on their surfaces following MBD adsorption. In addition, after contact with MBD, the porous texture was filled and vanished. Interaction with MBD changed the surface texture of the algal cell surface from smooth to rough and irregular. Meanwhile, tiny swellings were observed after contact with MBD.

#### 3.1.3. UV—Visible Spectral Examination

**Figure 4:**
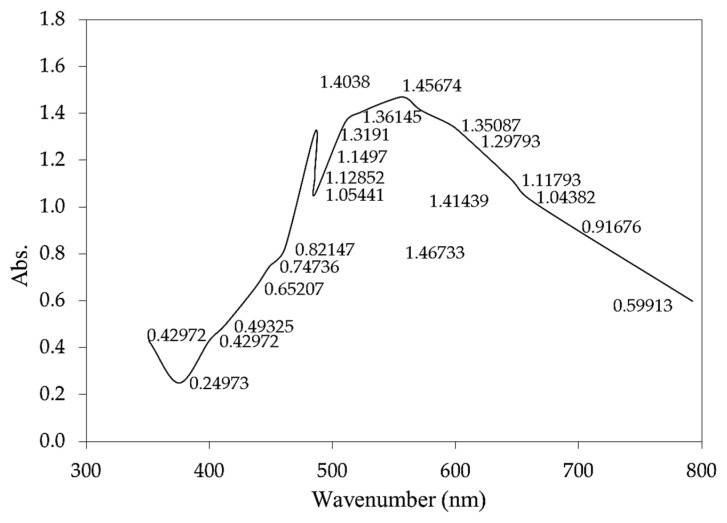
UV-Visible spectrum of *S. linifolium* extracts obtained with aqueous extract.

Spectral analysis in the ultraviolet (UV) ranges according to the sharpness of the peaks, the UV-VIS spectrum summary of *S. latifolium* extract was chosen between 200 and 800 nm, as shown in Figure 4. Flavonoids and their derivatives have unique absorption spectra. The spectral bands of flavonoids are typically absorption spectra with maximal values in the range of 300–360 nm [80]. The absorption maximum bands for anthocyanins are at 460–560 nm, while flavones and flavonols are around 310–370 nm, as seen in peaks (350, 375, 399, 461,485, 547, and 559 nm) [81,82]. Furthermore, the profile revealed that the compounds were separated at 608, 657, and 694 nm. The exact location and virtual intensities of these peaks provide a wealth of information about the flavonoids’ nature [83]. These peaks at 234–676 nm indicate the presence of phenolic and alkalidic chemicals in *S. latifolium*. The extract has some comparable alkaloid, flavonoids, and glycoside components when compared to the spectra of seeds and flowers [84,85]. The absorption spectra of *S. latifolium* seaweed extract revealed a broad absorbance range between 300 and 700 nm [86] with absorption maxima in the visible area of the solar spectrum at 412.5, 608, and 657 nm. The characteristic absorption of chlorophylls can be related to these absorption peaks [86,87].

#### 3.1.4. BET Characterization

To investigate any changes in the porosity of the sample under investigation, the specific surface area and pore volumes of the adsorbent were investigated as presented in Table 1. The *S. latifolium* isotherm displays quick N_2_ uptake at low pressures (P/P_0_ 0.01) and constant high adsorption with hysteresis at higher pressures. The surface area of the BET is estimated to be 111.65 m^2^/g. Because the pores operate as binding or receptor sites during the adsorption phase, this is critical for pollutant trapping. Additionally, the pore volume is 0.122 cc/g.

### 3.2. Study of Batch Adsorption Process

#### 3.2.1. pH

**Figure 5:**
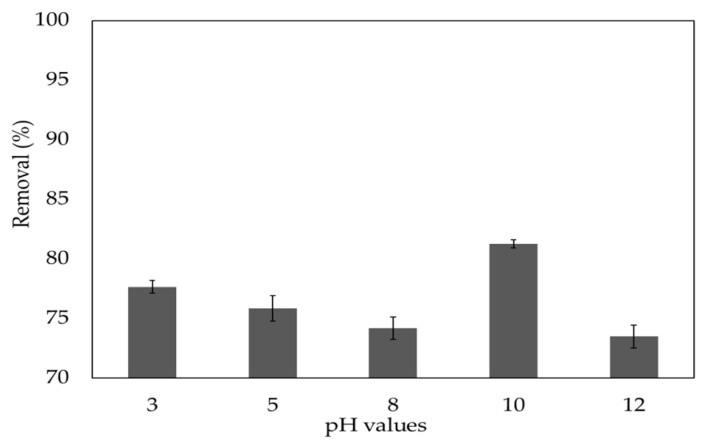
Impact of the initial pH on removal efficiency of methylene blue dye.

The pH value of the MBD solution has a significant effect on the adsorption mechanism because it affects both the adsorbent’s surface binding sites and the dye molecules’ ionization process. The net charge on the adsorbent is also pH-dependent since the adsorbent surface contains polymers with numerous unique functional groups such as phosphates, carboxyl, amino, and hydroxyl. In this work, the influence of pH was determined for pH values ranging from 3 to 12, as shown in Figure 5.

The *S. latifolium* can be employed in a wide pH range, and the rate of MBD removal increases as the pH value increases. When the pH was 10, the removal rate was 81.3%, demonstrating that the pH of the solution had an important influence on MBD adsorption. The presence of a positive charge on the nitrogen in the MB dye’s structure indicates that it is a basic and cationic dye. The surface charge on the adsorbent can be used to explain the influence of pH. However, the poor MBD adsorption has been seen at low pH values (pH 3), increasing the density of positive charge (protons) at surface biomass sites. The electrostatic repulsion that occurs between the MBD cations and the positive charges on the adsorbent’s surface explains this. The rate of negative surface charge of the biomass increases at a higher pH value (pH > 3), which electrostatically attracts cationic dyes [88], resulting in good MB adsorption on the surface of the adsorbent. Furthermore, the strong physical interactions (such as H-bonds) between both the adsorbate molecules (MBD) and various functional groups, namely hydroxyl, esters, alkynes, carbonyl, and carboxyl present in the phenols and flavonoids present in the noncellulosic cells of adsorbents of brown algal Sargassum, may describe the adsorption of MBD on *S. latifolium*.

The surface charge on the adsorbent, which is affected by the solution pH, is the most important factor in the adsorption of these positively charged dye groups on the surface of the adsorbent [89]. Methylene blue adsorption by dried *Ulothrix* sp. Biomass was examined by Doğar, et al. [90], who showed that the adsorption increased as the pH was increased.

#### 3.2.2. MDB Concentration

**Figure 6:**
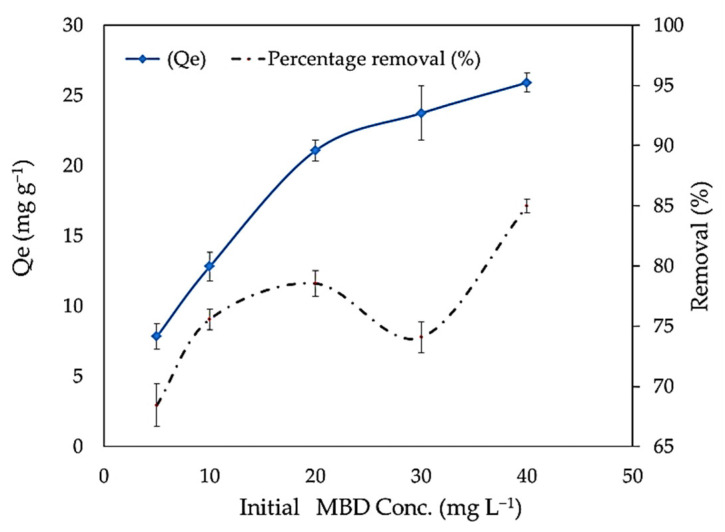
Impact of different methylene blue dye concentrations on adsorption capacity and removal percentages.

The initial dye concentration works as a forceful driving force, increasing the dye’s mass transfer resistance between the aqueous and solid phases to its limit. The effect of concentrations of MBD on the adsorption was studied at 5, 10, 20, 30, and 40 mg L^−1^ with the initial pH value of 10. Figure 6 shows the relationship between the percentage removals of MBD and adsorbent capacity (q_e_) and the equilibrium dye concentration in the liquid phase (Ce). When the MBD initial concentration is augmented from 5 to 40 mg L^−1^, the quantity of MBD adsorbed increases from 68.42% to 84.96%. In addition, the adsorption capacity is improved from 7.8 to 25.9 by increasing the initial dye concentration from 5 to 40 mg L^−1^ as shown in Figure 6. The total number of dye molecule collisions and macrophytes increases when the initial dye concentration is increased. As a result, increasing the initial dye concentration could accelerate the adsorption process [91]. Furthermore, it might be attributed to an increase in the driving force of the concentration gradient with the increase in the initial concentration [92]. This is in agreement with results reported by other studies [2].

#### 3.2.3. Sorbent Loading

**Figure 7:**
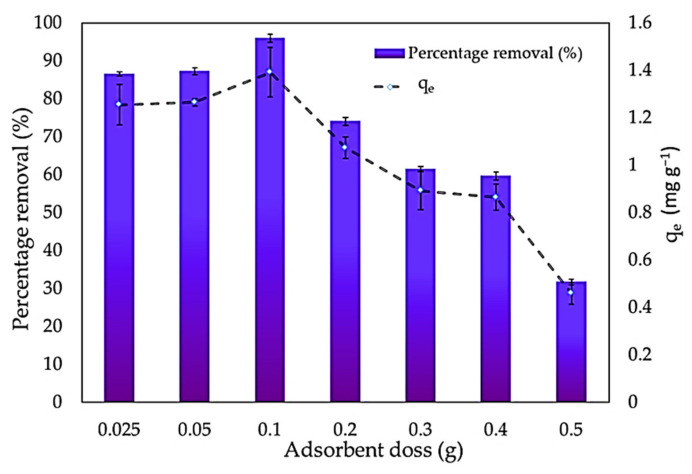
Impact of adsorbent dose on adsorption capacity and removal percentages of methylene blue dye.

At an MBD concentration of 10 mg L^−1^, the impact of the adsorbent amount on methylene blue removal was examined. In the MBD solution, *S. latifolium* concentrations varying from 0.025 to 0.5 g L^−1^ were incorporated. Figure 7 shows that when the adsorbent loading was raised from 0.025 to 0.1 g, the elimination effectiveness increased from 86.53 to 95.97 percent, then dropped after this value. The availability of additional active adsorption sites, as well as an increase in the adsorptive surface area, is the key reason for this. Furthermore, adsorption onto the adsorbent surface happens very quickly at higher adsorbent dosages. The amounts of MBD adsorbed per unit weight of seaweed were lowered when the adsorbent loading was augmented from 0.2 to 0.5 g. Increased biomass loading may lead to a decrease in the removal percentage value due to the complicated interaction of numerous factors, for example, solute availability, binding site interference, and electrostatic interactions [93]. At higher adsorbent dosages, the available MBD molecules are inadequate to cover all of the exchangeable sites on the adsorbent, resulting in the limited dye uptake. Additionally, as adsorbent mass increases, the amount of dye adsorbed q_e_ (mg g^−1^) decreases due to a split in the flow or concentration gradient between the solute concentration in the solution and solute concentration on the adsorbent surface [2].

#### 3.2.4. Contact Time

**Figure 8:**
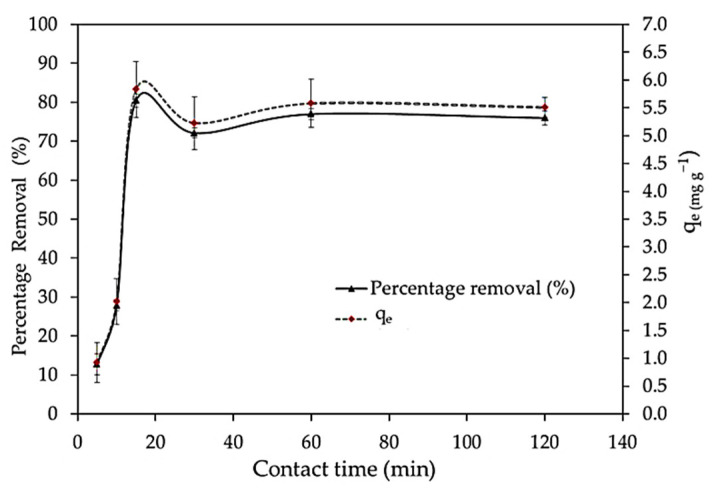
Impact of different contact times on adsorption percentages of the methylene blue dye.

The equilibrium adsorption of *S. latifolium* was measured at various contact time intervals to investigate the influence of different contact times on adsorption. The percentage of elimination efficiency increases as contact time increases (Figure 8). The result showed that the percentage of removal efficiency increased from 12.7% to 80.43% with increasing the heating time from 5 to 15 min, then decreased to 76% after the first and the second hour of contact time (Figure 8).

The rapid adsorption at the initial contact time is due to the availability of the positively charged surface of the *S. latifolium* for the adsorption of MBD. Moreover, it is clear that MBD ions adsorption mainly consists of two stages, an initial rapid stage related to the instantaneous external surface adsorption of MBD ions with the more available reactive groups, which allows quick binding of ions on the biomass [94], whereas fast adsorption equilibrium was reached in the first 15 min. This could be due to the high specific surface area of the adsorbent particles and the absence of internal diffusion resistance at this stage [95]. As these sites became progressively covered, the rate of adsorption decreased. The second stage is much slower, with gradual adsorption levels taking place and lasting until adsorbate ions adsorption attains equilibrium, which is attributed to the diffusion of dye ions into the adsorbent layers for binding on the inner reactive groups, with the aid of hydrophilic and swelling characteristics of the adsorbent material. Therefore, the initial adsorption uptake rate is increased while the second step is slowed down [96]. Additionally, Khan et al. [97] reported that the transfer of the dye from the solution phase into the pores of the adsorbent is the rate-controlling stage in batch experiments under rapid stirring circumstances. Likewise, Sheen [98] found that adsorption was rapid in the first 5 min of contact with an uptake of more than 90%, and equilibrium was reached in 60 min of agitation time as the binding sites became exhausted. As the binding sites became exhausted, the uptake rate slowed due to metal ions competing for the decreasing availability of active sites. According to the test results, the rest of the batch experiment’s shaking period was set at 120 min to ensure that equilibrium was achieved. This quick (or rapid) adsorption phenomenon is advantageous in applications because the short contact time effectively allows for a smaller contact equipment size, which has a direct effect on both the capacity and operation cost of the process.

#### 3.2.5. Temperature

**Figure 9:**
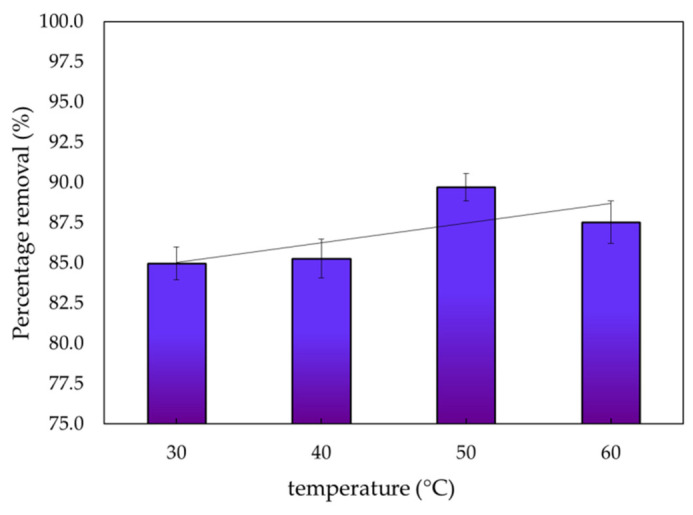
Impact temperature on the percentages of adsorption of methylene blue dye.

The equilibrium uptake of dye into the biomass of *S. latifolium* was also influenced by the temperature parameter (Figure 9). The uptake of MBD onto the dried biomass was found to be most effective at 50 °C, with a removal percentage of 89.71%. With a decrease in temperature, adsorption decreased. The viscosity of the dye-containing solution is reduced as the temperature rises, allowing more dye molecules to diffuse through the external boundary layer and into the internal pores of the adsorbent particles [99]. Temperature also has a major influence on dye degradation, first by directly changing the chemistry of the pollutant, and then by influencing its physiology.

### 3.3. Equilibrium Adsorption

Sorption equilibrium is reached when the concentration of solutes adsorbent onto matches the amount of associated development. When this happens, the equilibrium solution concentration remains constant. By graphing the concentration of the solid phase versus the concentration of the liquid phase, the equilibrium adsorption isotherm will be seen. The Freundlich, Langmuir, Harkins–Jura, Halsey, Henderson, Smith, and Tempkin models were used to analyze equilibrium data.

#### 3.3.1. Freundlich Isotherm

**Figure 10:**
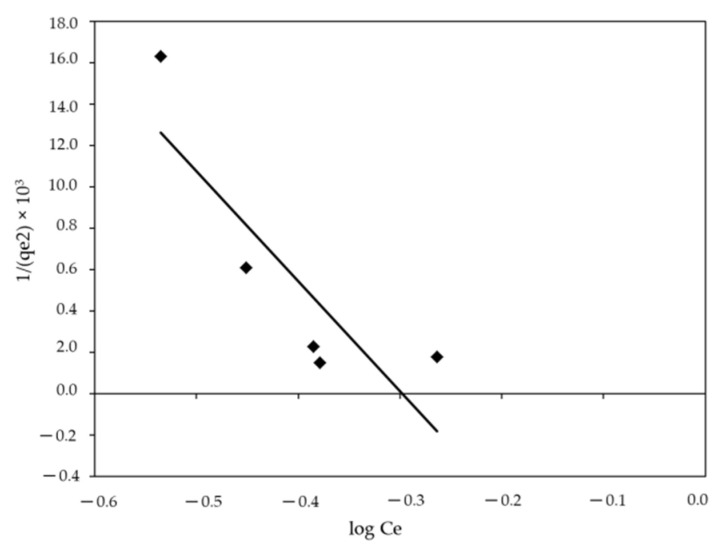
Linear Freundlich isotherm for MBD sorption.

The Freundlich adsorption isotherms with the correlation coefficient are shown in Figure 10 and Table 2.

The R^2^ (0.906) and 1/n (1.115) results indicate that adsorption isotherms and monolayer coverage on the adsorbent surface are applicable, based on the discovered linear relationships. The value of 1/n, also known as the heterogeneity factor, describes the deviation from sorption linearity as follows: if 1/n = 1, the adsorption is linear, and the dye particle concentration does not affect the division between the two stages. Chemical adsorption occurs when 1/n is less than 1, whereas cooperative adsorption occurs when 1/n is more than 1, which is more physically favorable and involves strong contacts between the adsorbate particles [100]. In this study, the values of factor “1/n” are more than 1, indicating that using this isotherm equation to properly perform the physical sorption mechanism on an exterior surface is preferable. The adsorbent could be used as a low-cost adsorbent for MBD removal due to its outstanding monolayer adsorption properties. Additionally, the Freundlich isotherm equation can be used to describe heterogeneous systems and can be applied to adsorption on heterogeneous surfaces with the interaction between adsorbed molecules.

#### 3.3.2. Langmuir Isotherm

**Figure 11:**
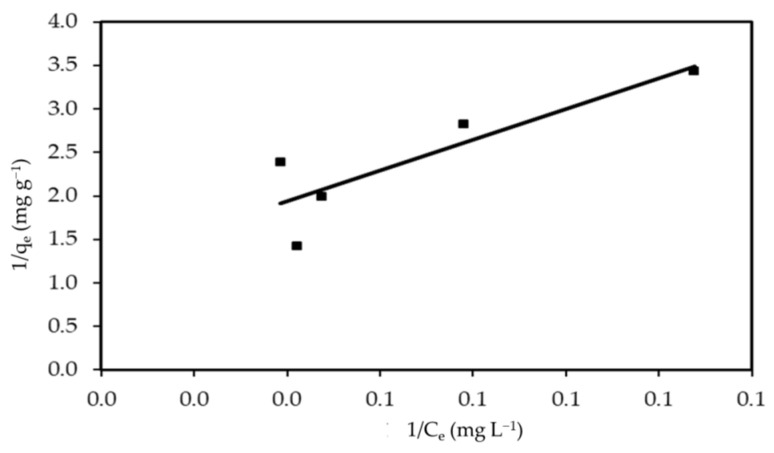
Linear Langmuir isotherm for MBD sorption.

The regression data and adsorption parameters obtained are shown in Figure 11 and Table 2. The Langmuir equation’s linear form has the advantage of pointing to the high success rate of using algal biomass for dye removal. R^2^ = 0.751 for MBD shows the monolayer coverage of MBD on the outer surface of the sorbent, in which the mechanism of adsorption occurs uniformly on the reactive section of the surface. The sorbent shows that the Langmuir model does not apply to the tested system. The maximum adsorption capacity (q_max_) is 0.819 mg g^−1^, which corresponds to complete monolayer coverage on the surface. The adsorption capacities (q_max_) of various adsorbents for MBD are compared in Table 3. q_max_ of *S. latifolium* is significantly higher than those of other adsorbents. Due to its low cost, reusability, and high adsorption capacity, R_L_ can also be used to express the fundamental features of a Langmuir isotherm, which is determined from the relation of Hall et al. [101]:

(23) $$R_{L}=\frac{1}{1+\;\mathrm{bCi}}$$

The types of equilibrium isotherms are related to the R_L_ values; for R_L_ > 1, an unfavorable process dominates, but for R_L_ = 1, a favorable mechanism is available [109]. Table 2 shows that the values of R_L_ in this study ranged from zero to one (0.969), indicating that MBD adsorption on algal biomass was favorable.

#### 3.3.3. Tempkin Isotherm

**Figure 12:**
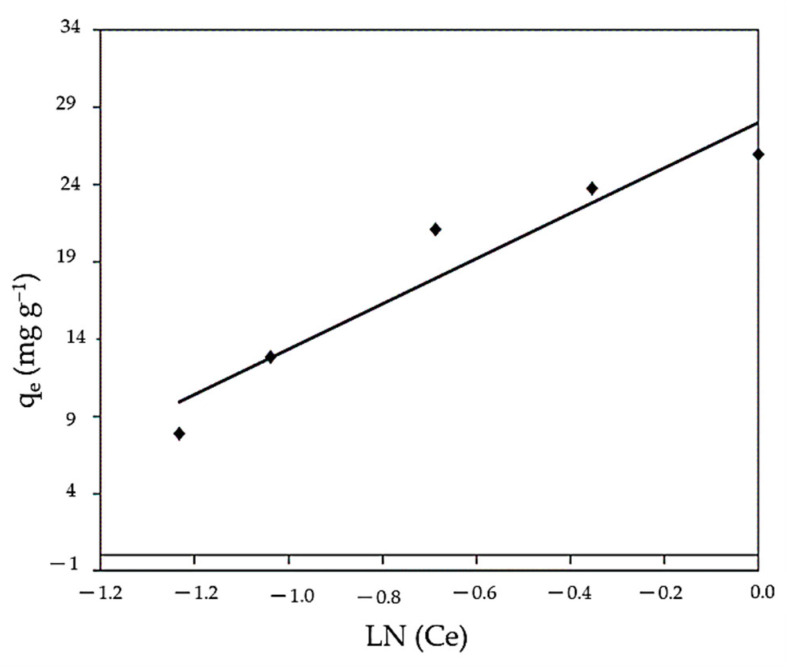
Linear Tempkin model for MBD sorption.

The Tempkin isotherm model posits that the heat of adsorption of all molecules reduces linearly rather than logarithmically, with coverage due to the interaction between adsorbate and adsorbent. From the Tempkin model (Figure 12 and Table 2), the following values were obtained: A = 2.47 L g^−1^, B = 14.67 J mol^−1^, and b_T_ = 168.8. The correlation coefficient value (R^2^ = 0.916) is high and demonstrates a satisfactory well to the experimental data where the sorption process of MBD of the algal biomass does obey the Tempkin isotherm. Additionally, it assumes that the heat of adsorption of all the molecules in the layer decreases linearly with coverage due to adsorbate–adsorbate repulsions and that the adsorption is a uniform distribution of maximum binding energy [110].

#### 3.3.4. Harkins–Jura Isotherm

**Figure 13:**
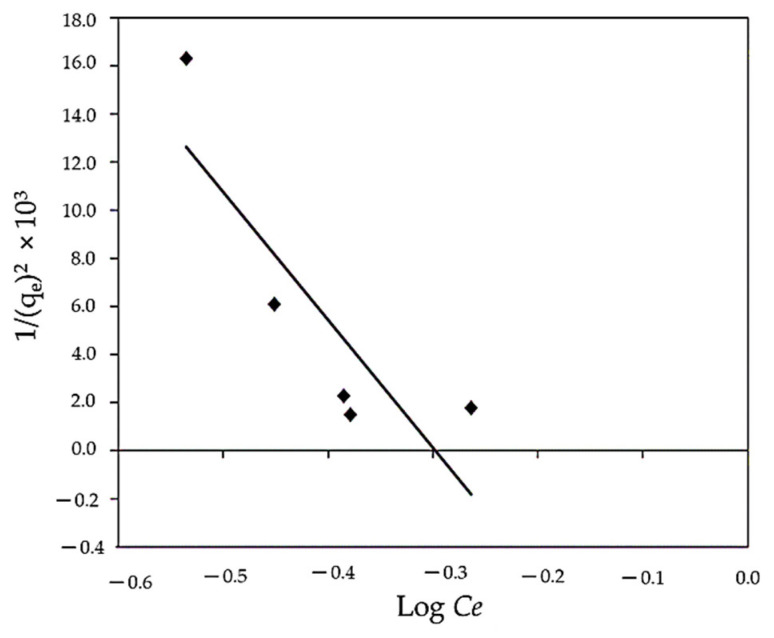
Linear Harkins–Jura isotherm for MBD sorption.

As shown in Figure 13, the Harkins–Jura adsorption isotherm can be written as Equation (8), which can be solved by plotting 1/q_e_ vs. log C_e_. The presence of a heterogeneous pore distribution and multilayer adsorption can be represented by the Harkins–Jura model. The range of isotherm constants and correlation coefficients is R^2^ = 0.719 (Table 2). This may indicate that the Harkins–Jura model is less applicable.

#### 3.3.5. Halsey and Henderson Isotherm

**Figure 14:**
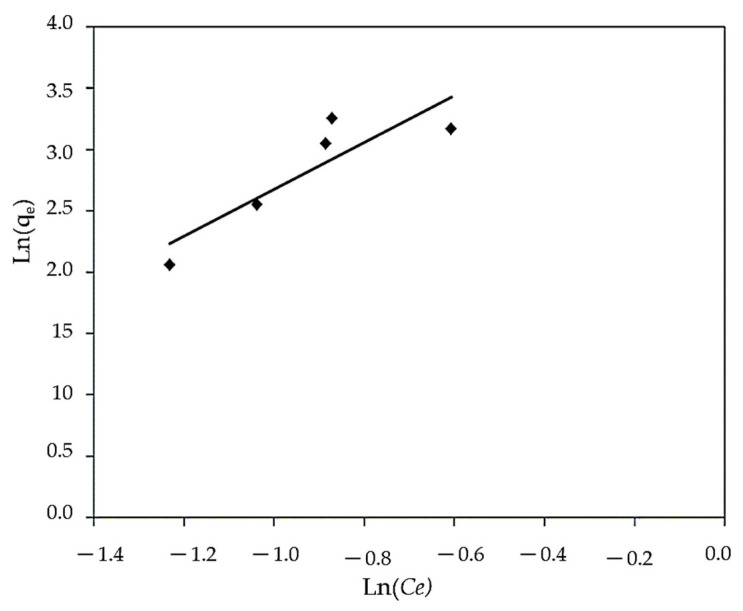
Linear Halsey isotherm for MBD sorption.

**Figure 15:**
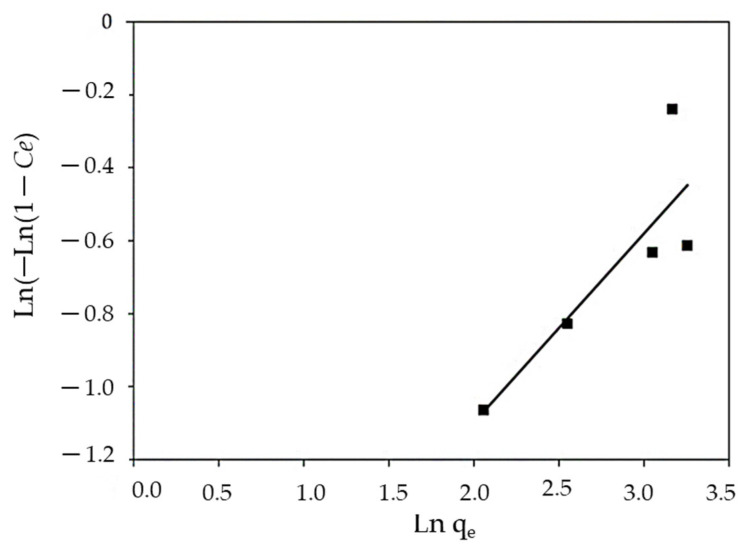
Linear Henderson isotherm for MBD sorption.

Figure 14 and Figure 15 show the Halsey and Henderson adsorption isotherms, respectively. The Halsey isotherm shows that the correlation coefficient was R^2^ = 0.768, while Henderson shows the regression coefficient value of R^2^ = 0.735. The results obtained from Halsey and Henderson show that both models are small and did not demonstrate a satisfactory response to the experimental data where the sorption process of MBD onto algal biomass was concerned.

#### 3.3.6. Smith Isotherm

**Figure 16:**
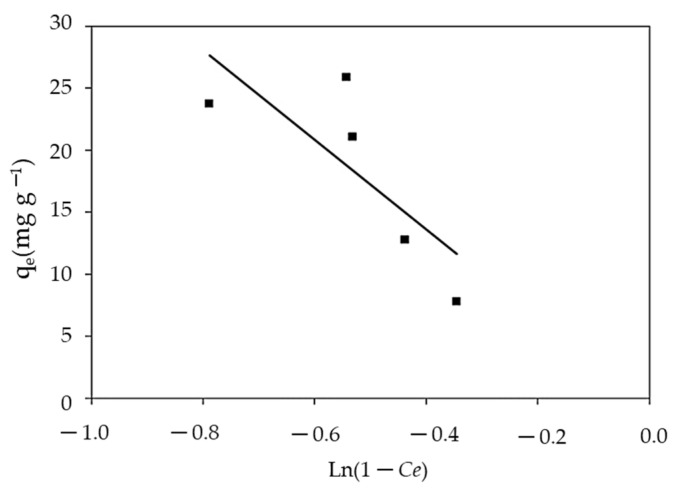
Smith isotherm for MBD sorption.

Smith’s model is useful in explaining the adsorption isotherm of biological substances, such as cellulose and starch, in addition to being appropriate for heterosporous solids and multilayer adsorption [111]. The Smith model can be solved by the plot of q_e_ vs. Ln (1 − Ce) as presented in Figure 16 and Table 2. The sorption isotherms were accurately reproduced by Smith models across the entire range of water activity. However, Smith’s equation was not effective in describing the isotherms of MBD on algal biomass because the model gave lower R^2^ values (0.605).

#### 3.3.7. Error Functions Examination for Best-Appropriate Isotherm Model

Traditional linear regression methods for calculating isotherm parameters appear to provide a satisfactory fit to the experimental data. The R^2^ is determined using the linear versions of the isotherm equations, but it eliminates the isotherm curve error [71]. To assess the fit of the isotherm equations to the experimental data, several error functions of non-linear regression were employed to establish the constant model parameters, and they were compared to those determined from less precise linearized data fitting [112]. Several error functions, such as the hybrid fractional error (Hybrid), the average percentage errors (APE) equation, the Chi-square error (X^2^) equation, the sum of the squares of the errors (ERRSQ), and the sum of absolute errors (SAE), were applied to determine the most-fit model for the investigational data (EABS). Table 4 summarizes the data gathered from various error functions. From the observed data, the best appropriate isotherm models are Halsey, Henderson, Freundlich, Langmuir Harkins–Jura, Tempkin, and Smith. However, the error functions studied gave variable results for each isotherm model and the comparison between the isotherm models should be focused on each error function separately. The (EABS) equation demonstrates that all the isotherm models can be applied to the investigational data except the Harkins–Jura, Tempkin, and Smith isotherms. Analysis of Table 4 shows that the Halsey, Henderson, Freundlich, and Langmuir models have smaller errors in almost all of the cases and that the Harkins–Jura, Tempkin, and Smith models did not show high accuracy. In this case, the Freundlich isotherm model can be more useful for describing the adsorption process of MB by *S. latifolium* adsorbent.

### 3.4. Kinetic Models

**Figure 17:**
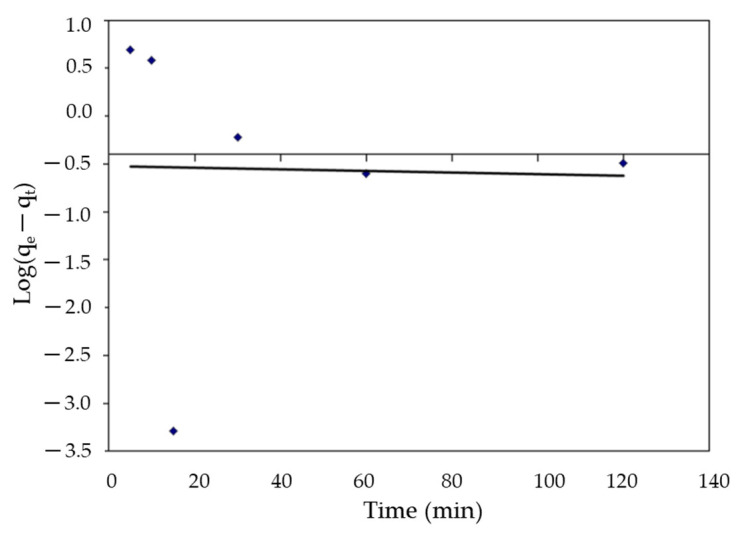
The pseudo-first-order equation for the elimination of MBD onto *S. latifolium*.

**Figure 18:**
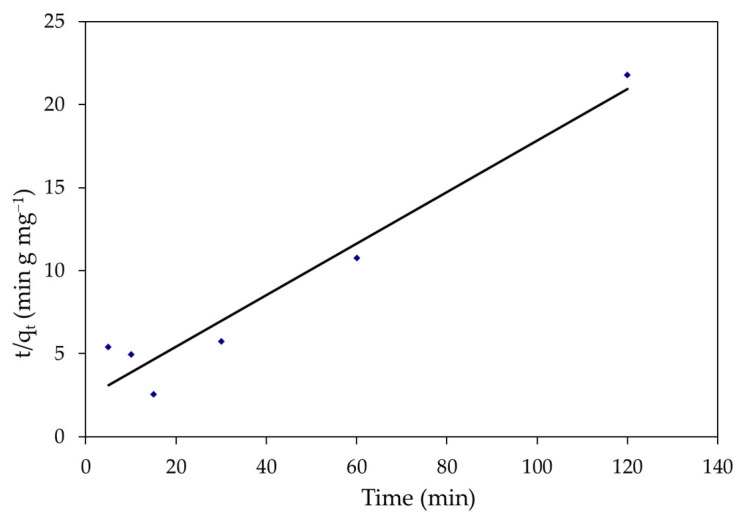
The pseudo-second-order for the elimination of MBD onto *S. latifolium*.

**Figure 19:**
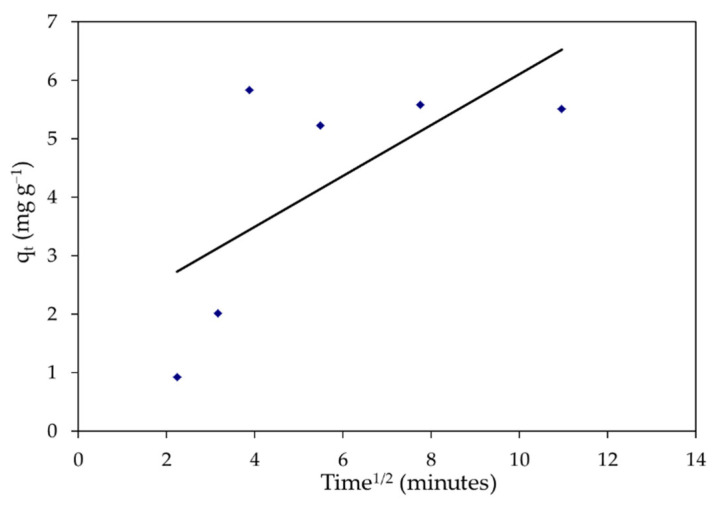
The intraparticle diffusion equation for the elimination of MBD onto *S. latifolium*.

Kinetic analysis was performed using pseudo-first and second-order models, as well as the intraparticle diffusion model, to evaluate the adsorption processes of MBD on *S. latifolium*. These models are commonly used to describe the sorption of dyes and other pollutants on solid sorbents, as well as to evaluate their applicability. For the adsorption of a solute from a liquid solution, the Lagergren rate equation is one of the most extensively used adsorption rate equations. The linear regressions are shown in Figure 17, Figure 18 and Figure 19. In addition to the intraparticle diffusion model, Table 5 shows the coefficients of the pseudo-first and second-order adsorption kinetic models.

The linearized form of the plot log (q_e_ − q_t_) versus time (t) as shown in Figure 17 showed a poor pseudo-first-order linear regression coefficient (R^2^) value of 0.001. The slope of the plot is used to calculate the rate constant of pseudo-first-order adsorption. Furthermore, the pseudo first-order kinetic model predicted a significantly lower equilibrium adsorption capacity (q_e_ = 3.30) than the experimental value (q_e_ = 4.181), indicating the model’s impermissibility.

On the other hand, the intercept and slope of the plots of t/q_t_ vs. t, were used to compute the second-order rate constant, k_2_, and the equilibrium adsorption capacity, qe. Figure 18 depicts pseudo-second-order kinetic graphs. In addition, the linear regression coefficient (R^2^) value for the pseudo-second-order kinetic model was 0.943, indicating that the model is applicable, as shown in Table 5. The (q_e_) computed value was close to the experimental data, hence a pseudo-second-order model was fitted. Generally, the kinetic studies displayed that the adsorption of MBD was followed by pseudo-second-order kinetics models and the sorption process was controlled through the chemisorption process. Similar results have been reported for the adsorption of organic pollutants [113,114]. Furthermore, understanding the adsorption system’s dynamic behavior is critical for process design. The removal of MBD by adsorption on biomass was shown to be quick for the first 15 min, but thereafter became slow as contact duration increased. The dye molecules migrate from the bulk solution to the surface of the sorbent and penetrate through the boundary layer to the surface of the sorbent, where adsorption and intra-particle diffusion actually occur, according to the intra-particle diffusion mechanism for the sorption system of MBD removal from its aqueous phase [115]. The intercept is C, and the slope directly predicts the rate constant K_dif_, as illustrated in Figure 19. The form of Figure 16 reveals that straight lines do not pass through the origin and that the removal of MBD on *S. latifolium* has a poor correlation (0.446), reflecting the fact that the intraparticle diffusion model does not suit the experimental results. Because the resistance to exterior mass transfer increases as the intercept increases, the value of the C factor provides information about the thickness of the boundary layer.

### 3.5. Thermodynamic Studies

Table 6 displays the values for ΔG°, ΔH°, and ΔS°. The reaction is spontaneous and endothermic, as evidenced by the positive values of ΔH° and ΔS°. The negative values of ΔG° also represent the process’s spontaneity. The values of ΔG° get increasingly negative as the temperature rises, indicating that the process is becoming more spontaneous. Physical binding forces may be involved in the process, as evidenced by the low value of ΔH°. The range of ΔG° values for physisorption is between −20 and 0 kJ mol^−1^, but the range for chemisorption is between −80 and −400 kJ mol^−1^. According to the activation energy estimations in this work, MBD sorption onto the marine algae *S. latifolium* occurred via a physisorption mechanism.

### 3.6. Treatment of Real Dye Effluent

To check the efficiency of *S. latifolium* as an adsorbent for the removal of MBD from industrial effluents, simulated water was prepared as a control to study the influence of biomass on the adsorption process, and optimization conditions (at an acidic pH 10, 0.1 g of adsorbent after 120 min at a higher temperature of 50 °C) were used. Due to the low quantities of MBD, a large amount of MBD was applied to achieve a final concentration of 40 mg L^−1^. Industrial effluent samples were obtained from a factory in Al-Mahla Al-kobra, Egypt, which specializes in dyeing and printing. To track the percentage of the dye combination eliminated from the real dye effluents, the UV–vis spectra of the simulated water and effluents treated with *S. latifolium* were recorded at 665 nm. The results confirmed that changing the type of water affected the largest removal, with simulated water having the least impact on the sorption process, with a percentage removal of 89.15%. Real wastewater, on the other hand, contains very high concentrations of interfering ions from a variety of contaminants, which had a major impact on the removal effectiveness of MBD, with a percentage removal of 65.9%.

The novelty in this research lies in different values of the optimization process, mathematical models, and the highest suitable model being achieved by using error function tests such as a fractional error hybrid, average percentage error, sum squares of errors, nonlinear chi-square analysis, and the sum of absolute errors. To describe the elimination of the MBD’s effects on *S. latifolium*, error functions were proposed. This study is unique in that it highlights the possibility of integrating the use of marine algae biomass into the removal of MBD from aqueous environments in accordance with circular economic principles. Thus, using a simple and abandoned, readily available, low-cost, cheap, and environmentally friendly bio-material experimental procedure, the *S. latifolium* material, which can be more efficiently used to retain MBD, is obtained from a low-performance adsorbent material, such as marine brown algae biomass. Therefore, *S. latifolium* was chosen to prepare the adsorbent based on the above parameters. A step was taken to prepare the adsorbent, which was then utilized to remove the MBD from the water.

## 4. Conclusions

In the current study, brown seaweed, *S. latifolium,* was applied as an adsorbent for the elimination of methylene blue dye (MBD) from an aqueous solution. Initial pH, initial MBD concentration, adsorbent dose, and adsorption temperature all had an impact on adsorption. The highest removal of MB was 95.97% under optimal operating conditions, which included an initial MBD concentration of 40 mg L^−1^, a temperature of 50 °C, and a pH of 10. Increases in the adsorbent dose and starting MBD concentrations increased MBD removal effectiveness. Furthermore, MBD removal using *S. latifolium* is also influenced by the presence of functional groups such as carbonyl, hydroxyl, amino, carboxylate groups, and other natural compounds that support ions binding like lignins, cellulose, and lipids. Additionally, SEM analysis demonstrates the ability of *S. latifolium* biomass to adsorb and remove MBD from an aqueous solution. Moreover, the absorption bands for photosynthetic pigments, such as flavonoids and their derivatives, were found in the UV-visible spectra of seaweed. In addition, the quality of *S. latifolium* was also assessed using BET characterization, which shows that *S. latifolium* has a greater surface area. MBD adsorption on *S. latifolium* followed Halsey, Henderson, Harkins–Jura, Freundlich, Tempkin, Smith, and Langmuir isotherms revealed in the research. *S. latifolium* was found to be a good adsorbent for removing MBD from wastewater through the adsorption process.

## Figures and Tables

**Table 1:** Textural properties of *S. latifolium* as determined by N_2_ adsorption.

Surface Area Results (m^2^/g)					Total Pore Volume	Average Pore Size	Average Particle Radius
Single Point BET	Multipoint BET	Langmuir Method	BJH Adsorption	BJH Desorption			
100.78	111.65	178.40	67.87	58.486	0.12 cc/g	2.19 nm	1.22 + 001 nm

**Table 2:** Factors of isotherm models from non-linear and linear solvation.

Isotherm Model	Isotherm Parameter	Value
Freundlich	1/n	1.115
	K_F_ (mg^1–1/n^L^1/n^g^−1^)	31.6
	R^2^	0.906
Langmuir	Q_max_ (mg g^−1^)	0.819
	b (L mg^−1^)	0.068
	R_L_	0.969
	R^2^	0.751
Tempkin	A_T_	2.47
	B_T_	14.67
	b_T_	168.88
	R^2^	0.916
Harkins–Jura	A	0.02
	B	0.3
	R^2^	0.719
Halsey	1/n_H_	1.912
	K_H_	11
	R^2^	0.768
Henderson	1/n_h_	0.519
	K_h_	0.119
	R^2^	0.735
Smith	W_bs_	0.768
	Ws	36.01
	R^2^	0.605

**Table 3:** Comparison of higher monolayer sorption of MBD onto different adsorbents materials.

Adsorbent	Capacities (mg g^−1^)	Reference
**Banana peel**	0.124	[102]
**Cotton stalk**	11.6	[103]
**Wheat shells**	16.6	[104]
**Coir pith carbon**	5.9	[105]
**Wheat shells**	16.6	[104]
**Activated date pits**	12.9	[106]
**Cereal chaff**	20.3	[107]
**Fly Ash**	0.0727	[108]
***S. latifolium***	0.819	This study

**Table 4:** The values of the five various error assessments of isotherm models for adsorption.

Isotherm Model	Hybrid	APE%	X^2^	ERRSQ	EABS
**Freundlich**	5.453	2.035	1.854	4.707	13.017
**Langmuir**	363.42	13.66	83.58	305.55	87.40
**Harkins–Jura**	202.700	10.203	46.621	170.425	65.273
**Halsey**	0.151	0.278	0.035	0.127	1.781
**Henderson**	0.605	0.557	0.139	0.508	3.565
**Smith**	391.312	14.177	90.002	329.004	90.692
**Tempkin**	359.232	13.583	82.623	302.033	86.895

**Table 5:** The comparison of the first order, second-order, and intraparticle diffusion for adsorption rate constants.

Model	Parameter	Value
**First-order Kinetic**	q_e_ (calc.)	3.30
	K_1_	2.07
	R^2^	0.001
**Second-order Kinetic**	q_e_ (calc)	6.44
	K_2_	0.01
	R^2^	0.936
	q_e_ (exp.)	4.18
**Interparticle Diffusion**	K_dif_	1.747
	C	0.44
	R^2^	0.45

**Table 6:** Thermodynamic factors of the sorption of IV MB 2R onto *S. latifolium*.

Temperature (°C)	ΔG° (kJ mol^−1^)	ΔH° (kJ mol^−1^)	ΔS° (J mol^−1^)
30	−6.35	35.205	−0.131
40	−6.62		
50	−7.86		
60	−7.54		

## Data Availability

The data that support the findings of this study are available from the authors upon reasonable request.
